# Blocking GARP-mediated activation of TGF-β1 did not alter innate or adaptive immune responses to bacterial infection or protein immunization in mice

**DOI:** 10.1007/s00262-021-03119-8

**Published:** 2022-01-01

**Authors:** Mélanie Gaignage, Xuhao Zhang, Julie Stockis, Olivier Dedobbeleer, Camille Michiels, Perrine Cochez, Laure Dumoutier, Pierre G. Coulie, Sophie Lucas

**Affiliations:** 1 de Duve Institute, Université Catholique de Louvain, avenue Hippocrate 74, B1.74.04, 1200 Brussels, Belgium; 2 Walloon Excellence in Life Sciences and Biotechnology (WELBIO), Wavre, Belgium

**Keywords:** GARP, TGF-β1, Monoclonal antibody, Intestinal bacterial infections, *Citrobacter rodentium*, Protein immunization

## Abstract

**Abstract:**

Transmembrane protein GARP binds latent TGF-β1 to form GARP:(latent)TGF-β1 complexes on the surface of several cell types including Tregs, B-cells, and platelets. Upon stimulation, these cells release active TGF-β1. Blocking TGF-β1 activation by Tregs with anti-GARP:TGF-β1 mAbs overcomes resistance to PD1/PD-L1 blockade and induces immune-mediated regressions of murine tumors, indicating that Treg-derived TGF-β1 inhibits anti-tumor immunity. TGF-β1 exerts a vast array of effects on immune responses. For example, it favors differentiation of T_H_17 cells and B-cell switch to IgA production, two important processes for mucosal immunity. Here, we sought to determine whether treatment with anti-GARP:TGF-β1 mAbs would perturb immune responses to intestinal bacterial infection. We observed no aggravation of intestinal disease, no systemic dissemination, and no alteration of innate or adaptative immune responses upon oral gavage of *C. rodentium* in highly susceptible *Il22r*^*−/−*^ mice treated with anti-GARP:TGF-β1 mAbs. To examine the effects of GARP:TGF-β1 blockade on Ig production, we compared B cell- and T_H_ cell- responses to OVA or CTB protein immunization in mice carrying deletions of *Garp* in Tregs, B cells, or platelets. No alteration of adaptive immune responses to protein immunization was observed in the absence of GARP on any of these cells. Altogether, we show that antibody-mediated blockade of GARP:TGF-β1 or genetic deletion of *Garp* in Tregs, B cells or platelets, do not alter innate or adaptive immune responses to intestinal bacterial infection or protein immunization in mice. Anti-GARP:TGF-β1 mAbs, currently tested for cancer immunotherapy, may thus restore anti-tumor immunity without severely impairing other immune defenses.

**Précis:**

Immunotherapy with GARP:TGF-β1 mAbs may restore anti-tumor immunity without impairing immune or inflammatory responses required to maintain homeostasis or host defense against infection, notably at mucosal barriers.

**Supplementary Information:**

The online version contains supplementary material available at 10.1007/s00262-021-03119-8.

## Introduction

Transforming growth factor-β1 (TGF-β1) is a potent immunosuppressive cytokine that plays an important role in the maintenance of immune tolerance [1, 2]. Most cells, including immune cells, produce TGF-β1 in a latent, inactive form, in which the mature TGF-β1 dimer is non-covalently associated with the latency-associated peptide (LAP) [3, 4]. Only a few cell types are able to activate the cytokine, by releasing mature TGF-β1 from LAP and allowing its binding to the TGF-β receptor. Dendritic cells and epithelial cells can activate latent TGF-β1 deposited in the extracellular matrix via binding of integrins αVβ8 or αVβ6, respectively, to RGD motifs in LAP [5, 6]. We and others showed that Tregs, B cells, and platelets activate latent TGF-β1 presented on their surface by a transmembrane protein called GARP [7–9].

Activation of TGF-β1 from GARP:(latent)TGF-β1 complexes on Tregs requires integrin αVβ8 [10]. We recently developed monoclonal antibodies against GARP:TGF-β1 complexes that block TGF-β1 activation and immunosuppression by human and mouse Tregs [11, 12]. We showed that anti-GARP:TGF-β1 mAbs overcome resistance to PD1/PD-L1 blockade and induce immune-mediated regressions of tumors in mice. In addition to blocking Treg immunosuppression and restoring anti-tumor immunity, anti-GARP:TGF-β1 mAbs could exert unwanted side effects, owing on one hand to their ability to block TGF-β1 activation from non-Treg GARP-expressing cells, and on the other hand to the pleiotropic functions exerted by TGF-β1 in immunity, including adaptive immunity. Notably but non exclusively, TGF-β1 is known to induce switch to IgA production in B cells, and differentiation of naïve CD4^+^ T cells into T_H_17 effectors in presence of IL-6 or into peripheral Tregs (pTregs) in presence of IL-2 [13-15]. T_H_17, pTregs and IgA-producing B cells are adaptive immune effectors playing important roles in the establishment and maintenance of balanced immune responses at epithelial barriers. The use of blocking anti-GARP:TGF-β1 mAbs for the purpose of cancer immunotherapy could therefore perturb immunity against bacterial infections in the intestine.

*Citrobacter rodentium* is a natural murine bacterial pathogen causing intestinal infection, inflammation, and disease that closely resembles disease caused by enteropathogenic *Escherichia coli* and enterohemorrhagic *E. coli* in humans. Oral gavage of *C. rodentium* in WT mice causes infection and inflammation limited to colon and caecum, which are rapidly controlled by the immune system, preventing severe intestinal disease [16, 17]. Production of IL-22 is required to protect the host against development of severe colitis [18]. In the early phase of infection, IL-22 is produced by innate immune cells such as group 3 innate lymphoid cells. The cytokine is crucial to limit bacterial expansion, notably by inducing production of RegIIIβ and RegIIIγ antimicrobial peptides by epithelial cells [19, 20]. In later phases, IL-22 is also produced by CD4^+^ T cells, including T_H_17 cells. In addition to T_H_17 cells, adaptive immune responses against *C. rodentium*, which are required to clear the infection [21, 22], also imply T_H_1 cells and B cells producing pathogen-specific IgGs.

Here, we examined whether anti-GARP:TGF-β1 mAbs could perturb innate or adaptive immune responses at mucosal barriers, using oral gavage of *C. rodentium* in WT or highly susceptible *Il22r*^*−/−*^ mice as a model of intestinal bacterial infection. We also examined whether the absence of GARP:TGF-β1 complexes would alter T cell- or B cell- responses against protein immunization in mice carrying Treg-, B cell- or platelet-specific deletions of the *Garp* gene.

## Methods

### Mice

All mice were bred at the SPF animal facility of the UCLouvain. Cell type-specific *Garp* KOs and WT littermates were obtained by crossing *Lrrc32*^*tm1.1Hfuj*^ mice with *B6.129 (Cg)-Foxp3*^*tm4(YFP/icre)Ayr/J*^, or *Tg(Pf4-icre)Q3Rsko, or Cd79a*^*tm1(cre)Reth*^*/EhobJ* mice. *Il22ra1*^*−/−*^ (*Il22r*^*−/−*^) mice were generated at the de Duve Institute [23]. Mice were maintained in an SPF animal facility at temperatures between 20 and 24 °C, HR between 40 and 65%, and day–night cycles of 12 h–12 h. All animal studies were performed in accordance with national and institutional guidelines for animal care, under permit number 2017/UCL/MD/019 from the UCLouvain.

### Antibodies

Clone 58A2 is a monoclonal mouse IgG2a antibody that binds mouse GARP:TGF-β1 complexes and blocks active TGF-β1 production by mouse cells in vitro [12]. Three to four biweekly intra-peritoneal (*i.p.*) injections of 250 µg of 58A2 mAb in combination with anti-PD1 were previously shown to exert anti-tumor effects in tumor-bearing mice [12]. Here, mice received two weekly *i.p.* injections of 400 µg of 58A2. Clone 1D11 is a monoclonal mouse IgG1 antibody that neutralizes active TGF-β [1, 2, and 3] (BioXcell).

### C. rodentium infections

*C. rodentium* strain DBS100 (kindly provided by M. Chamaillard, Pasteur Institute, Lille, France) was cultured overnight in LB media at 37 °C. Concentration of bacteria in the cultures was assessed by measuring absorbance at 600 nm and converting into colony-forming units (CFU). Inoculation of *C. rodentium* (10^9^ CFU in 200 µl of PBS) was performed by oral gavage in 3-month-old mice. One day before infection and 6 days after, 400 µg of anti-GARP:TGF-β1 (clone 58A2) or anti-TGF-β (clone 1D11) mAbs were injected *i.p.*. Mice were monitored daily for weight change, and sacrificed at the time point indicated in the figures, or if weight loss was > 20% by comparison today 0.

### Protein immunization

To measure Ig responses, 3-month-old mice were injected *i.p.* with 100 μg ovalbumin (OVA, Sigma) or 30 μg Cholera Toxin B subtype (CTB, Enzo Life Science) emulsified in 100 μl of Imject® Alum solution (Thermofisher) on day 0, and 100 μg OVA or 30 μg CTB in PBS on day 9. Mice were bled on day 16 to measure OVA- or CTB- specific Igs in the serum. As indicated in the figures and their legends, some mice received 400 µg of anti-GARP:TGF-β1 *i.p.* on day -1 and 6. To measure T_H_ cell responses, 3-month-old mice were injected sub-cutaneously (*s.c.*) with 100 μg OVA emulsified in 100 μl of Complete Freund’s Adjuvant (CFA, Thermofisher) on day 0, then sacrificed to collect spleens on day 14.

### Tissue collection and Histology

Colons were collected after sacrifice. Five mm-long terminal fragments were used for RT-qPCR analyses. For histological analyses, colons were placed in a Swiss roll shape, soaked in 10% formalin for 24 h then embedded into paraffin. 7 µm-sections were stained with hematoxylin and eosin. Histopathological scoring was adapted from previous reports [24, 25], by measuring lymphocyte infiltration, goblet cell- and crypt- damage, and colonic hyperplasia to attribute a colitis score (0: no colitis; 1: scattered inflammatory cells in the lamina propria, less than 25% of Goblet cell depletion, less than 25% of crypt thickness increase; 2: increased numbers of inflammatory cells in the lamina propria, less than 50% of Goblet cell depletion, less than 50% of crypt thickness increase 3: confluence of inflammatory cells extending into the submucosa, less than 75% of Goblet cell depletion, less than 75% of crypt thickness increase; 4: transmural extension of the infiltrative inflammatory cell severe colitis, 100% of Goblet cell depletion, 100% of crypt thickness increase).

### Bacteria and CFU counts

Fresh fecal samples were collected, weighed, and homogenized in cold sterile PBS (1 ml/100 mg of feces). Bacterial DNA was extracted using QIAamp DNA Stool Mini Kit (Qiagen). Copy numbers of the *C. rodentium Espb* gene were measured by qPCR with the following primer set: 5′- CGTCAGCAGCCTTTTCAGCTA -3′, and 5′- ATGCCGCAGATGAGACAGTTG -3′ and in 20 µl reaction volumes containing Takyon Master Mix (Eurogentec) using StepOnePlus device (Thermofisher) with standard Thermal cycling parameters (95 °C for 3′; 50 cycles of 95 °C for 10’’ and 60 °C for 60″).

Livers were collected, weighed and homogenized, and titrated in PBS. Series of liver homogenate dilutions were spread on LB semi-solid culture medium and incubated at 37 ℃ overnight. Bacterial colonies were counted to determine CFUs and normalized to the weight of the livers.

### Mixed lymphocyte cultures

15 days after immunization with OVA in CFA, spleens were collected and CD4^+^ T cells were sorted by MACS (Miltenyi Biotech) with anti-mouse CD4^+^ beads. 2 × 10^5^ CD4^+^ cells were seeded with irradiated syngeneic adherent cells pulsed with OVA. Adherent cells were obtained by coating 1 × 10^6^ splenocytes in a 96-well flat-bottomed plate for 1.5 h, then removing non-adherent cells by washing with PBS, and pulsed with OVA (50 µg/ml) for 2 h, before irradiation (30 Gy from a ^137^Cs source). After 96 h of mixed lymphocyte culture, supernatants were collected to measure cytokines.

### RNA extraction and RT-qPCR

Colonic tissues were disrupted with the Tissue Lyser (Qiagen), total RNA was isolated using Nucleospin Mini Columns (Macherey Nagel), and reverse transcribed into cDNA (Thermofisher). qPCR was performed in a StepOnePlus device (Applied Biosystems) in 20 µl reaction volumes containing Takyon Master Mix (Eurogentec), cDNA, and primers. Thermal cycling parameters were either fast conditions (95 °C for 3′; 50 cycles of 95 °C for 3’’and 60 °C for 30’’) or standard conditions (95 °C for 3′; 50 cycles of 95 °C for 10’’ and 60 °C for 60’’) depending on the amplicon size. The *b-actin* gene (primers obtained from Eurogentec; forward strand: 5′-ATTGCCGACAGGATGCAGAA-3′; reverse strand: 5′-GTCATACTCCTGCTTGCTGA-3′; Taqman probe: 5′-TCAAGATCATTGCTCCTCCTGAGC-3′) was used to normalize relative gene expression. Primers were obtained from Eurogentec or IDT. The target genes included *Il17a* (forward: 5′-GCTCCAGAAGGCCCTCAG-3′; reverse: 5′-CTTTCCCTCCGCATTGACA-3′; Taqman probe: 5′-ACCTCAACCGTTCCACGTCACCCTG-3′), *Ifng* (forward: 5′-TCAAGTGGCATAGATGTGGAAGAA-3′; reverse: 5′-TGGCTCT GCAGGATTTTCATG-3′; Taqman probe: 5′-TCACCATCCTTTTGCCAGTTCCTCCAG-3′), *RegIIIb* (forward: 5′-CTACTGCCTTAGACCGTGCTTTC-3′; reverse: 5′-GAGTCTTCACATTTTGTCCCTTGTC-3′; Taqman probe: 5′-GTGAAGTTGCCCTATGTCTGC-3′), *RegIIIg* (forward: 5′-GAGTGGAGCAATGCTGATGTGATG-3′; reverse: 5′-GGGATCTTGCTTGTGGCTAGG -), *Il6* (forward: 5′-CAGAGTCCTTCAGAGAGATACAGAAA-3′; reverse: 5′-TCCAGCTTATCTGTTAGGAGAGCATT-3′), *Il4* (forward: 5′-GAACGAGGTCACAGGAGAAGG-3′; reverse: 5′-GGACTCATTCATGGTGCAGCTTA-3′; Taqman probe: 5′-CCTCACAGCAACGAAGAACACCACAG-3′) and *Il22* (forward: 5′-GCTGCCCGTCAACACCC-3′; reverse: 5′-CTGATCCTTAGCACTGACTCCTC-3′; Taqman probe: 5′- TGAGGTGTCCAACTTCCAGCAGCCA-3′).

### ELISA

Total serum IgG, IgM, and IgA were measured according to manufacturer instructions using IgG, IgM, and IgA ELISAs kits (Thermo Scientific). To measure *C. rodentium-* specific serum Igs, bacteria were lysed by sonication in PBS, *C. rodentium* proteins were enriched with bacterial protein extraction reagent (Thermo Scientific), then coated overnight at 4 °C (10 µg/ml, 100 µl) prior to incubation with various dilutions of mouse sera as indicated in the figures. Similarly, to measure OVA- and CTB- specific Igs, OVA (15 µg/ml, 100 µl) or CTB (4 µg/ml, 100 µl) were coated overnight at 4 °C on microtiter plates. *C. rodentium-*, OVA- or CTB- specific IgGs, IgMs, and IgAs were measured using detection antibodies from the IgG, IgM, and IgA ELISA kits. Cytokines in culture supernatants were also measured by ELISA (murine IFNγ ELISA, R&D Systems; murine IL-17a ELISA, with antibodies described in [26]). Absorbance readings were made at 450 nm, using a 96-well plate spectrophotometer with GloMax Discover (Promega).

### Flow cytometry

Splenocytes were stained with antibodies against surface markers (CD4, B220, CD41, and GARP) in the presence of a viability dye (eBioscience) and anti-CD16/32 to block FcγRs using a standard protocol. Tregs were stained with anti-Foxp3 using the eBioscience™ FOXP3/Transcription Factor Staining Kit (Invitrogen). Analyses were performed on a FACS LSR Fortessa flow cytometer (DIVA, BD Biosciences) and data were computed using the FlowJo software (Tree Star).

### Statistical analyses

Statistical analysis was performed with Prism 5 (Graphpad Software) using non-parametric tests (Mann–Whitney), One-way ANOVA (Bonferroni multiple comparison post-tests), and Log-rank Test for survival curves. *P*-value is shown if it is lower than 0.5.

## Results

### Anti-GARP:TGF-β1 mAbs do not aggravate intestinal disease caused by C. rodentium infection

**Fig. 1:**
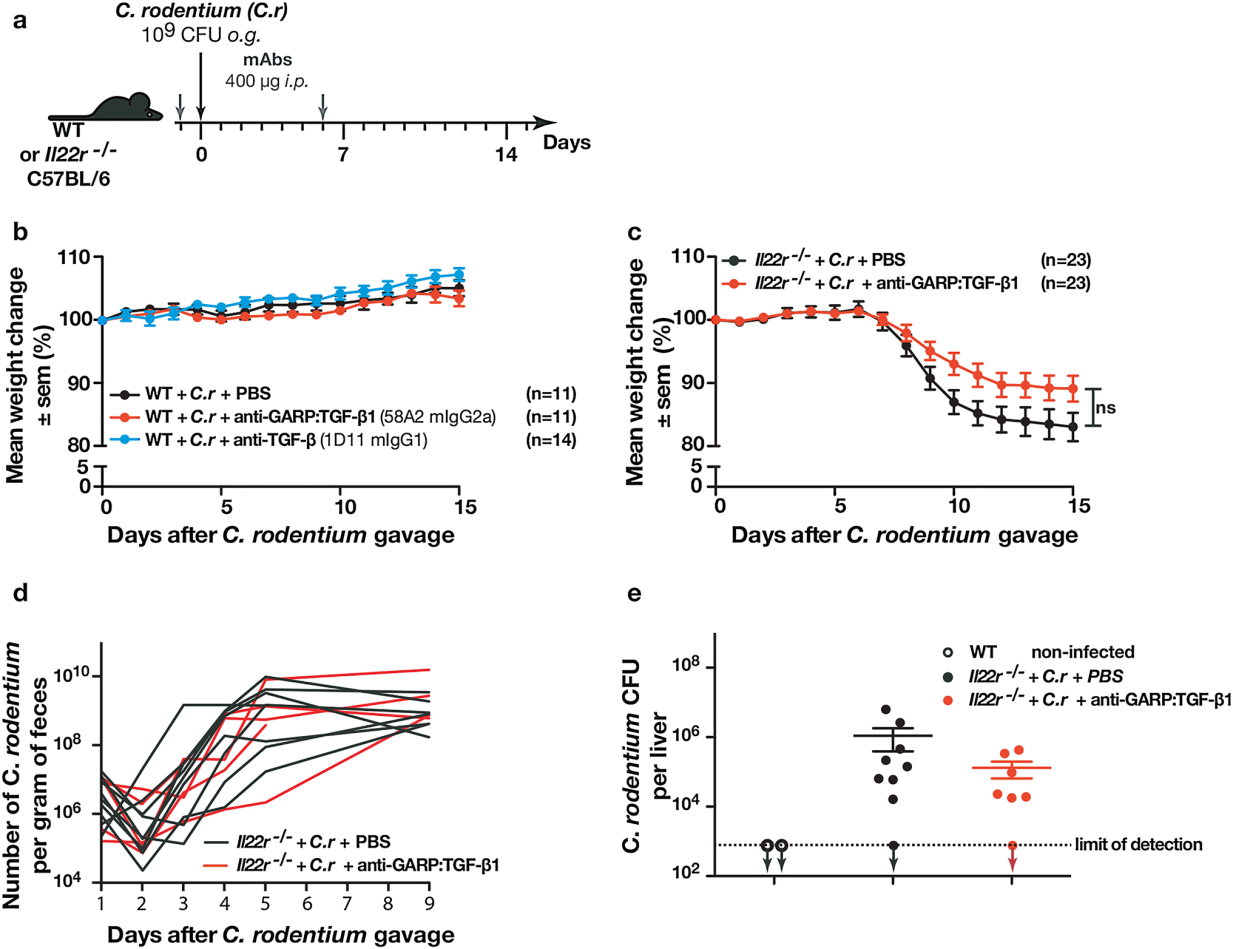
Antibody-mediated blockade of TGF-β1 produced from GARP:TGF-β1 complexes does not aggravate intestinal disease induced by *C. rodentium* infection. **a** Schematic representation of the experimental design. Three-month-old C57BL/6 wild-type (WT B6) or *Il22r*^*−/−*^ mice received *i.p.* injections of PBS, anti-GARP:TGF-β1 or anti-TGF-β mAbs 1 day before, and 6 days after oral gavage with *C. rodentium* (*C.r*). **b-c** Mice were monitored daily for weight loss. Data pooled from 2 to 3 independent experiments. Data points represent mean weight change per group ± sem. n = number of mice per group. Statistical analysis was performed by ANOVA with a Bonferroni post-test. **d** *C. rodentium* numbers were evaluated by quantification of *espB* gene copy number in the feces by qPCR. Each line represents values measured in one mouse. **e** *C. rodentium* CFUs in the livers collected 9 days after oral gavage. Each data point represents the value measured in one mouse. Statistical analysis was performed with a Mann–Whitney unpaired t-test. No statistically significant difference was observed between infected mice treated with PBS or anti-GARP:TGF-β1 (*P* > 0,05)

Infection of wild-type C57BL/6 (WT) mice by oral gavage of *C. rodentium* is efficiently controlled by innate and adaptive immune responses, which prevent severe intestinal disease and weight loss [16, 17]. To determine whether TGF-β1 blockade could alter this control, we administered anti-GARP:TGF-β1, anti-TGF-β, or PBS one day before and six days after the oral gavage (Fig. 1a). Whereas the anti-GARP:TGF-β1 mAb (clone 58A2) blocks activation of latent TGF-β1 at the surface of GARP expressing cells such as Tregs [12], anti-TGF-β mAb (clone 1D11) neutralizes active TGF-β1, β2, and β3, whichever their cellular source. No obvious symptom and no weight loss were observed, and all mice survived in all groups, indicating that TGF-β signals are not required to prevent severe intestinal disease in WT mice (Fig. 1b and supplementary Fig. 1a).

IL-22 signaling is crucial in the early phase of host defense against intestinal infection. In contrast to WT mice, *Il22*^*−/−*^ and *Il22r*^*−/−*^ mice are highly susceptible to *C. rodentium* infection, which causes severe epithelial damage in the intestine, weight loss, systemic bacterial burden, and high mortality in these mice [19, 27]. We thus tested whether TGF-β1 blockade with anti-GARP:TGF-β1 mAbs would increase the severity of disease in these mice. Mice receiving control PBS injections started to lose weight 8 days after infection and had lost 17 ± 8% (mean ± sem) of their initial weight by the end of the experiment on day 15 (Fig. 1c). Only 30% of the mice survived until the end of the experiment (supplementary Fig. 1b). Injections of anti-GARP:TGF-β1 mAbs did not exacerbate weight loss, which was even slightly, although not significantly, less pronounced than in PBS-injected mice (11% ± 8%). In line with this, more than 65% of mice receiving anti-GARP:TGF-β1 mAbs survived until the end of the experiment (supplementary Fig. 1b). Histological analyses of colons collected 9 days after gavage confirmed epithelial damage and inflammation, which were not more severe in mice that had received anti-GARP:TGF-β1 mAbs (supplementary Fig. 1c). We used qPCR to measure *C. rodentium* numbers in the feces at multiple time points after oral gavage (Fig. 1d). After an initial drop on day 2, *C. rodentium* numbers per gram of feces started to increase on day 3, to reach a maximum of 10^8^–10^10^ on day 9. No significant difference was observed in mice receiving anti-GARP:TGF-β1 mAbs (Fig. 1d). We also measured *C. rodentium* CFU in liver homogenates, to evaluate systemic bacterial burden on day 9. No significant difference was observed in *Il22r*^*−/−*^ mice that had received anti-GARP:TGF-β1 by comparison to PBS (Fig. 1e).

Taken together, these results indicate that anti-GARP:TGF-β1 mAbs do not reduce control of bacterial proliferation nor does it aggravate the severity of disease induced by *C. rodentium* infection in WT mice or in highly susceptible *Il22r*^*−/−*^ mice.

### Anti-GARP:TGF-β1 mAbs do not modify innate and adaptive immune responses to intestinal C. rodentium infection

**Fig. 2:**
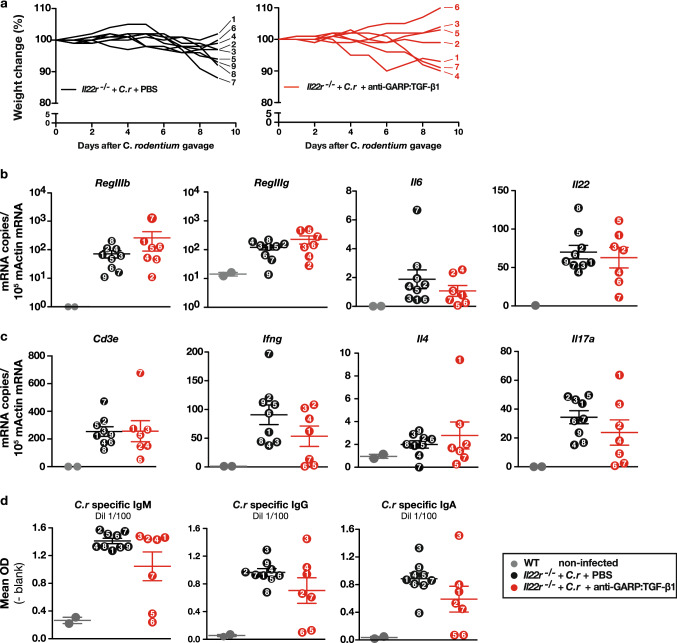
Antibody-mediated blockade of TGF-β1 produced from GARP:TGF-β1 complexes do not impair innate or adaptive immune responses against intestinal *C. rodentium* infection. Three-month-old *Il22r*^*−/−*^ mice received *i.p.* injections of PBS or anti-GARP:TGF-β1 mAbs 1 day before, and 6 days after oral gavage with *C. rodentium*. Mice were monitored daily for weight loss and sacrificed on day 9, to collect colons and sera. **a** Weight change in individual mice. Each line represents one mouse, with individual mouse ID numbers indicated next to the corresponding line. **b-c** Expression of the indicated genes, normalized to *b-actin* expression, as measured by RT-qPCR in colon samples. **d** *C. rodentium*-specific IgM, IgG, and IgA, as measured in 1/100 dilutions of serum samples by ELISA. Data points show mean value (technical duplicates) in each individual mouse (mouse ID number is indicated within each data point). Horizontal bars represent mean ± sem per group. Data is representative of three independent experiments. Statistical analysis was performed with a Mann–Whitney unpaired t-test. No statistically significant difference was observed between infected mice treated with PBS or anti-GARP:TGF-β1 (*P* > 0,05)

Control and clearance of *C. rodentium* infection require both innate and adaptive immune responses [16, 28]. We tested whether anti-GARP:TGF-β1 mAbs could alter these responses in the intestines of highly susceptible *Il22r*^*−/−*^ mice. Mice received *C. rodentium* by oral gavage on day 0, and *i.p.* injections of anti-GARP:TGF-β1 or PBS on days -1 and + 6. Weights were measured daily (Fig. 2a), and sera and colons were collected 9 days after gavage.

We first measured intestinal levels of mRNAs encoding molecules implicated in innate responses. High susceptibility to *C. rodentium* in absence of IL-22 signaling occurs mostly because IL-22 is required to induce expression of antimicrobial peptides RegIIIβ and RegIIIγ in colonic epithelial cells [19]. Accordingly, we observed that *RegIIIb* and *RegIIIg* mRNAs were induced in the intestines of infected *Il22r*^*−/−*^ mice by comparison to non-infected mice, but to levels ± 10–100 times lower than in infected WT mice (Fig. 2b and supplementary Fig. 2). Nevertheless, anti-GARP:TGF-β1 mAbs did not reduce *RegIIIb* and *RegIIIg* expression in infected *Il22r*^*−/−*^ or WT mice (Fig. 2b, supplementary Fig. 2 and data not shown). Similar results were observed for expression of *Il6* and *Il22* itself, taken here as representative of innate immune responses (Fig. 2b).

We next measured intestinal expression of mRNAs encoding CD3ε and cytokines produced by helper T (T_H_) cells. As expected, expression of *Cd3e*, *Ifng,* and *Il17a* was clearly induced by comparison to non-infected mice, whereas that of *Il4* was not. Here again, anti-GARP:TGF-β1 mAbs did not significantly reduce expression of any of these T cell marker or T_H_ cell-derived cytokines by comparison to control PBS injections (Fig. 2c). Finally, we measured *C. rodentium*-specific IgM, IgG and IgA in 1/100 dilutions of sera by ELISA. Most mice had induced high levels of *C. rodentium*-specific IgM, IgG, and IgA, whether or not they had received anti-GARP:TGF-β1 mAbs (Fig. 2d). Interestingly, two mice that had received anti-GARP:TGF-β1 (mice 5 and 6) did not develop strong IgM, IgG, and IgA responses against the bacteria. They correspond to mice that did not develop severe colitis and weight loss (Fig. 2a) and showed only very minor inductions of T_H_1 and T_H_17 cytokine genes in the colons (Fig. 2c). Thus, they appear to correspond to mice that did not develop significant infection and consequent immune responses upon oral gavage with *C. rodentium*.

Taken together, these results indicate that neither innate nor adaptive immune responses against *C. rodentium* in highly susceptible *Il22r*^*−/−*^ mice are altered by anti-GARP:TGF-β1 mAbs. If anything, anti-GARP:TGF-β1 mAbs tend to protect *Il22r*^*−/−*^ mice against severe intestinal disease and mortality induced by *C. rodentium* infection, although differences by comparison to PBS controls were not always statistically significant (Fig. 1c, supplementary Fig. 1b, and Fig. 2a).

### Anti-GARP:TGF-β1 mAbs do not alter production of antigen-specific IgM, IgG, or IgA following protein immunization

**Fig. 3:**
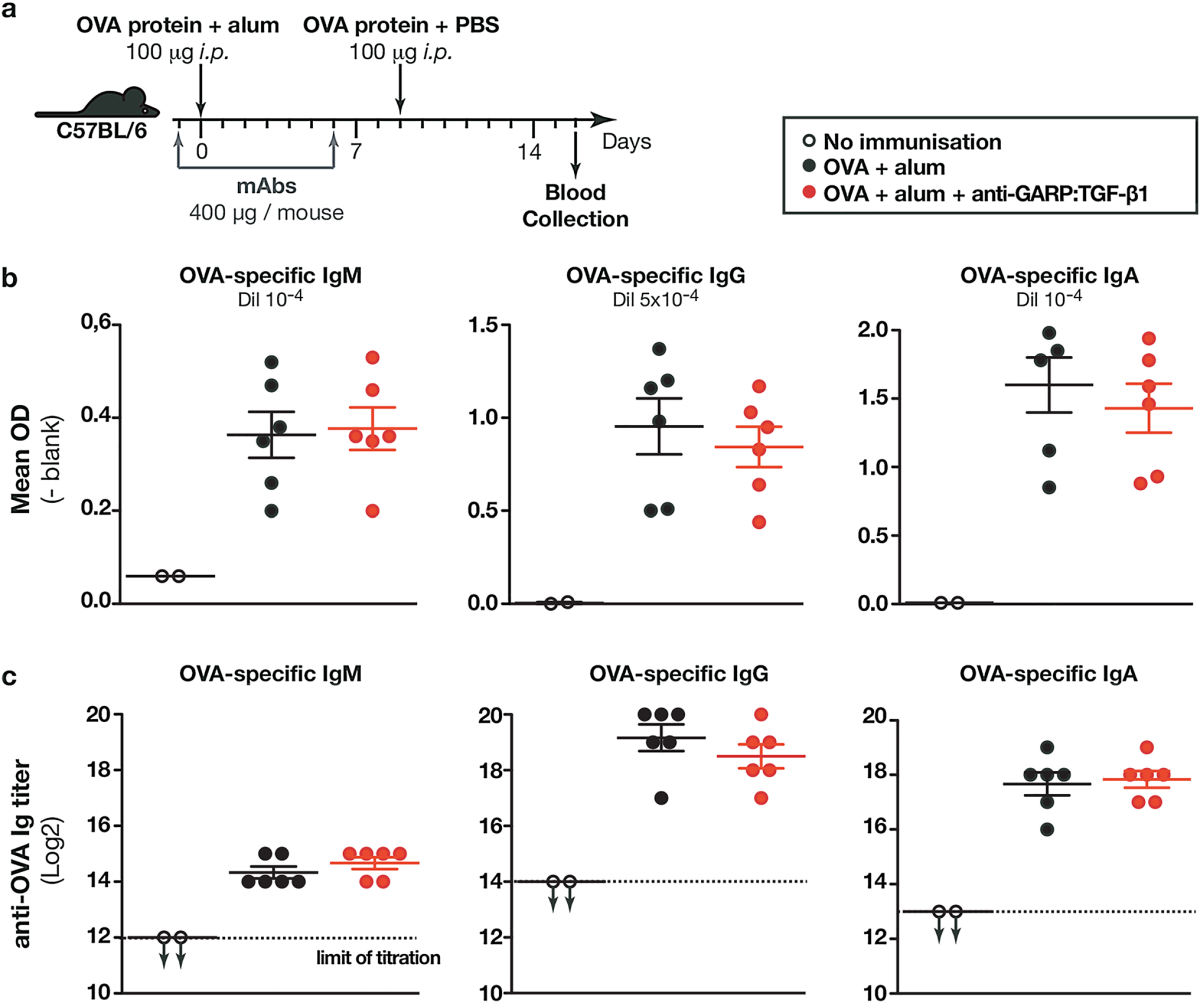
Antibody-mediated blockade of TGF-β1 produced from GARP:TGF-β1 complexes does not modify the amplitude of OVA-specific Ab responses upon immunization with OVA and alum. **a** Schematic representation of the experimental design. Three-month-old C57BL/6 mice were treated with mAbs 1 day before and 6 after *i.p.* OVA protein immunization with alum. After 9 days, OVA protein in PBS was injected *i.p.* as boost. Blood was collected on day 16 to measure OVA-specific Igs in sera by ELISA. **b-c** Data points represent values in individual mice. Horizontal lines indicate mean ± sem (n = 6 mice per group). Statistical analysis was performed with a Mann–Whitney unpaired *t-*test. No statistically significant difference was observed between OVA-immunized mice treated with PBS or anti-GARP:TGF-β1 (*P* > 0,05). Results shown are representative of two independent experiments

Our group previously reported that blocking anti-GARP:TGF-β1 mAbs impaired switch to IgA production in human B cells stimulated in vitro [9]. We thus sought to determine whether TGF-β1 production from GARP:TGF-β1 complexes could regulate Ig production and isotype switching in non-infectious immunization models that induce strong Ig responses in mice. We primed WT B6 mice with ovalbumin (OVA) in alum and boosted them in PBS on day 9. Mice also received PBS or anti-GARP:TGF-β1 injections 1 day before and 6 days after priming (Fig. 3a). Immunization induced abundant anti-OVA IgM, IgG, and IgA, none of which were reduced by anti-GARP:TGF-β1 mAbs (Fig. 3b-c).

### Genetic deletion of Garp in Tregs, B cells, or platelets does not modify production of antigen-specific IgM, IgG, or IgA following protein immunization

**Fig. 4:**
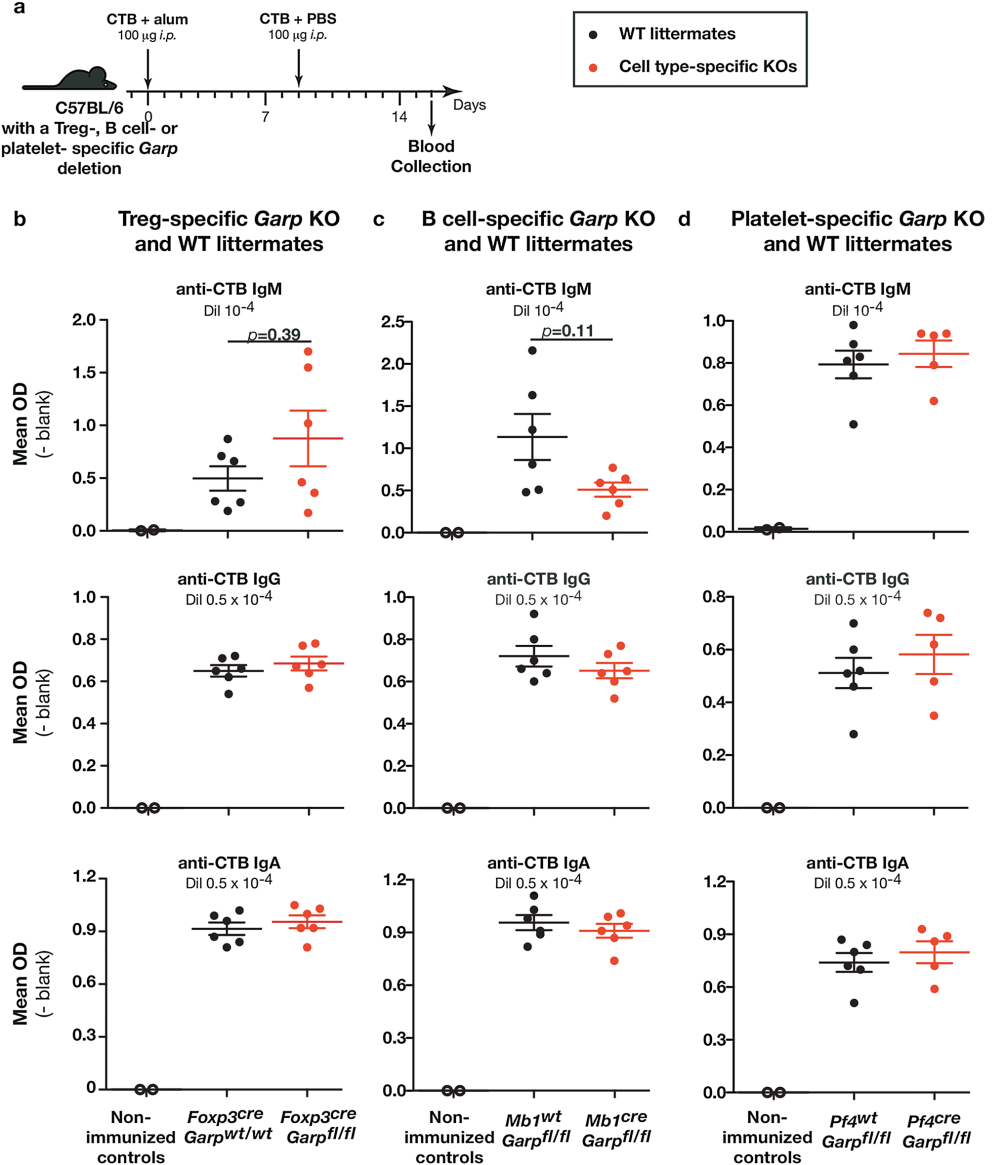
Genetic deletion of *Garp* in Tregs, B cells, or platelets does not modify CTB-specific Ig responses upon CTB immunization. **a** Schematic representation of the experimental design. Genetically modified C57BL/6 mice (3-month-old) were injected *i.p.* with CTB in alum on day 0, and CTB in PBS on day 9. Blood was collected on day 16 to measure CTB specific-Igs in serum by ELISA. **b-d** Data points represent values in individual mice. Horizontal lines show mean ± sem (n = 5–6 mice per group). Statistical analysis was performed with a Mann–Whitney unpaired *t*-test (to compare groups of CTB-immunized mice treated with PBS or anti-GARP:TGF-β1) and *p*-value is shown if it is lower than 0.5

To exclude cell-type-restricted effects of GARP:TGF-β1 blockade, we performed protein immunizations in mice carrying a Treg-, B cell- or platelet-specific deletion of the *Garp* gene. Complete and specific Cre-mediated deletion of *Garp* in the expected cell type is observed in each of the three mouse strains, namely *Foxp3*^*Cre*^ x *Garp*^*fl/fl*^, *Mb1*^*Cre*^ x *Garp*^*fl/fl*^, and *Pf4*^*Cre*^ x *Garp*^*fl/fl*^ mice (supplementary Fig. 3a). All cell-type-specific knock-out (KO) mice have serum levels of various Ig isotypes that are similar to those found in the corresponding WT littermates (Supplementary Fig. 3b). Treg-specific *Garp* KOs and WT littermates were immunized with OVA in alum as above. Immunization induced similar levels of anti-OVA IgM, IgG, and IgA in WT and KOs (supplementary Fig. 4). We also used another antigen, cholera toxin B (CTB), to immunize Treg- and other cell type-specific *Garp* KOs and WT littermates (Fig. 4a). CTB in alum is also known to induce high levels of antigen-specific IgM, IgG, and IgA [29, 30]. We confirmed that the absence of GARP on Tregs did not modify levels of anti-CTB IgM, IgG, and IgA in serum (Fig. 4b-c). *Mb1*^*Cre*^ x *Garp*^*fl/fl*^ and *Pf4*^*Cre*^ x *Garp*^*fl/fl*^ mice and their WT littermates were also immunized with CTB in alum and levels of serum anti-CTB IgM, IgG and IgA 16 days after priming were similar in B cell- and platelet- *Garp* KOs by comparison to their corresponding WT littermates (Fig. 4b-c). A trend towards slightly reduced anti-CTB IgM was observed in B cell-*Garp* KOs, but this difference was not statistically significant (Fig. 4c).

### Genetic deletion of Garp in Tregs, B cells, or platelets does not impair OVA-specific TH responses following OVA immunization

**Fig. 5:**
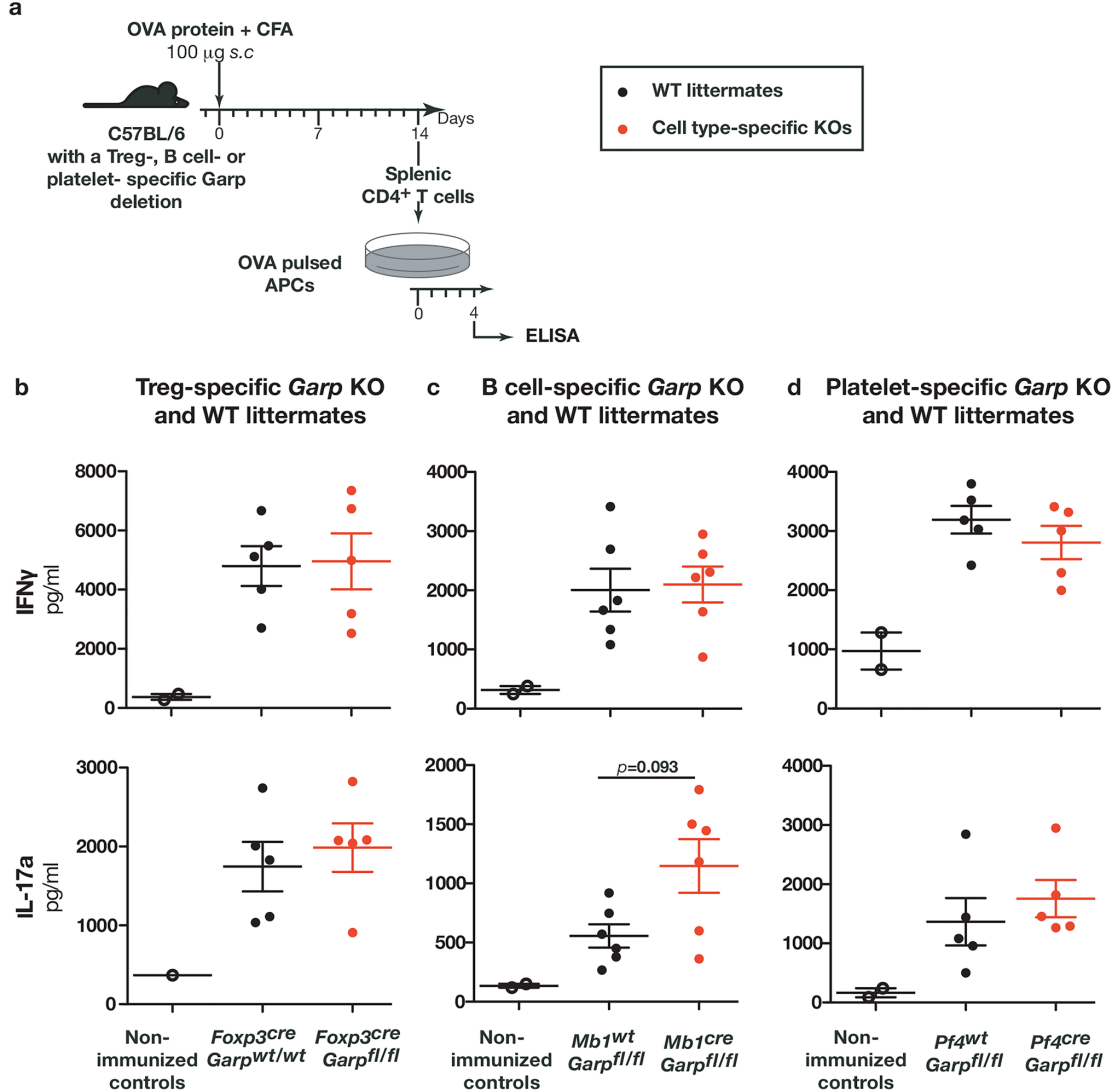
Genetic deletion of GARP in Tregs, B cells, or platelets does not modify OVA-specific T_H_ responses upon immunization with OVA and CFA. **a** Schematic representation of the experimental design. Genetically modified C57BL/6 mice (3-month-old) were injected *s.c.* with OVA protein and CFA on day 0. **B–c** 14 days after OVA-CFA immunization, spleen cells were collected and CD4^+^ cells were purified by MACS before incubation with adherent stimulating cells pulsed with OVA. After 4 days of co-culture, IFNγ and IL-17a production were measured by ELISA. Data in all panels are means ± sem (n = 5–6 mice per group) and are representative of two to three independent experiments Statistical analysis was performed with Mann–Whitney unpaired t-test (to compare the groups of CTB-immunized mice treated with PBS or anti-GARP:TGF-β1) and p-value is shown if it is lower than 0.5

We examined whether T_H_ cell differentiation is altered in cell type-specific *Garp* KOs upon protein immunization. *Garp* KOs and their WT littermates were immunized with OVA in Complete Freund’s Adjuvant (CFA). CFA is known to favor potent T_H_ response and notably T_H_17 responses [31], which could be impaired if TGF-β activation is reduced. Fourteen days after immunization, splenocytes were collected and re-stimulated in vitro with APCs pulsed with OVA to measure IL-4, IFNγ, and IL-17a production in the supernatants (Fig. 5a). Splenocytes from all mice immunized with OVA produced abundant IFNγ and IL-17a, but no IL-4. Importantly, no difference was observed between cell-type-specific *Garp* KOs and their corresponding WT littermates (Fig. 5b-d).

Altogether, our results indicate that TGF-β1 production from GARP:TGF-β1 complexes on Tregs, B cells, or platelets does not significantly impact antibody production and T_H_ cell differentiation following immunization with protein in vivo.

### Discussion

Our observations suggest that anti-GARP:TGF-β1 mAbs do not alter innate or adaptive immune responses against C. *rodentium* infection in mice. This may be considered reassuring with regards to the risk of increased susceptibility to intestinal infection and inflammation that could be associated with the use of anti-GARP:TGF-β1 mAbs for cancer immunotherapy. It also suggests that TGF-β1 derived from GARP-expressing cells does not play important role in regulating immune responses to bacterial infections at the level of mucosal barriers.

Zhang et al*.* reported that C. *rodentium* infection downregulates expression of TGF-β receptor chains I and II and of Smad2 in mouse colons [32]. They suggested that these downregulations promote inflammation and contribute to disease pathogenesis. Their observations could explain the absence of effect of anti-GARP:TGF-β1 mAbs observed here in mice infected with *C rodentium* if TGF-β1 signaling was already inhibited by the pathogen and did not participate in the immune responses.

Host defense against *C. rodentium* also involves adaptive T_H_17 responses [28]. T_H_17 differentiation depends on IL-23 and TGF-β1. IL-23-deficient (*p19*^*−/−*^) mice treated with anti-TGF-β1 mAbs developed severe colitis associated with a strong decrease of T_H_17 cells [33]. Backert et al*.* confirmed this observation with a TGF-β inhibitor in STAT3-deficient mice highly susceptible to *C. rodentium* infection [34]. Interestingly, we observed that anti-GARP:TGF-β1 mAbs did not alter *Il17a* mRNA expression in the colons of *C. rodentium*-infected *Il22r*^*−/−*^ mice, suggesting that active TGF-β1 released from GARP:TGF-β1 complex does not contribute to T_H_17 differentiation in this setting.

B cells and production of *C. rodentium*-specific IgGs are also required for complete clearance of *C. rodentium* [22]. Following C. *rodentium* infection, *Il22r*^*−/−*^ mice produced specific IgG, IgM, and IgA, and this production was not altered by administration of anti-GARP:TGF-β1 mAbs. This observation was unexpected. Indeed, we previously showed that blocking anti-GARP:TGF-β1 mAbs hampered isotype switching towards IgA by human B cells in vitro [9], suggesting that B cells could produce themselves the active TGF-β1 required for IgA secretion [15, 35]. However, our data here in mice showed that anti-GARP:TGF-β1 mAbs did not impede the anti-OVA IgA response in vivo. Moreover, anti-CTB IgA response was not impeded in any of the cell type-specific *Garp* KOs used, including B cell-specific *Garp* KO. Altogether, these results suggest that the active TGF-β1 required for IgA switching does not emanate from GARP-expressing cells, including B cells. This could be true when IgA switching occurs in vivo, upon immunization of mice with protein antigens. In our previous report using purified human B cells in vitro, IgA switching was observed after BCR stimulation with anti-IgM in the presence of CpG, anti-CD40L, and IL-21, but in the absence of any other cell types. In such experimental conditions in vitro, GARP-expressing human B cells may become a unique, predominant source of active TGF-β1 allowing IgA switching, even though they may not significantly contribute to switching in vivo.

Anti-GARP:TGF-β1 mAbs were shown to inhibit immunosuppression by human and mouse Tregs in vivo [11, 12]. Here, we observe no obvious impact of these mAbs in a model of bacterial intestinal infection in mice. We thus suggest that anti-GARP:TGF-β1 mAbs could be used to inhibit immunosuppression by Tregs in patients with cancer, without major risks of impairing immune responses to bacterial infection at mucosal barriers.

## Supplementary Information

Below is the link to the electronic supplementary material.

Supplementary file1 (DOCX 337 kb)
