# Supplementary material for: Blocking GARP-mediated activation of TGF-β1 did not alter innate or adaptive immune responses to bacterial infection or protein immunization in mice
Source: Cancer Immunol Immunother. 2022 Jan 1;71(8):1851–62. doi: 10.1007/s00262-021-03119-8 (PMC9294018; doi:10.1007/s00262-021-03119-8)
Supplement: Supplementary file 1 — Supplementary file1 (DOCX 337 kb) [file 262_2021_3119_MOESM1_ESM.docx]

**Supplementary Fig. 1 Antibody-mediated blockade of TGF-β1 produced from GARP:TGF-β1 complexes does not impair survival of mice after *C. rodentium* infection**

Three-month-old C57BL/6 mice were treated with mAbs (400 μg) 1 day before and 6 days after *C.rodentium* (10^9^ CFU) oral infection. **a** Wild-type and **b** *Il22r^-/-^* mice were monitored daily. Mice were sacrificed when weight loss was ≥ 20% of initial weight (on day 0). Percent surviving mice in two (**a**) or three (**b**) independent experiments are depicted. Statistical analyses were performed with a Log-rank test (*P<0.05). **c** Sections of colons of *Il22r^-/-^* mice collected 9 days after infection with *C.rodentium* were stained with H&E and analyzed for histological damages. On the left, representative sections illustrate crypt lengths (double black arrows), crypt damage/goblet cell depletion (red rectangles) and lymphocyte infiltration (green arrows). On the right, crypt lengths, crypt damage, goblet cell depletion and lymphocyte infiltration were scored on 0 (none) to 3 (severe) scale. Each data point represents the value measured in one mouse. Horizontal bars: mean ± SEM per group.

**Supplementary Fig. 2 *Il22r^-/-^* mice produce less RegIII antimicrobial peptides than WT mice after *C. rodentium* infection**

Three-month-old WT and *Il22r^-/-^* mice were orally infected or not with *C. rodentium*. *Il22r^-/-^* mice received i.p. injections of PBS or anti-GARP:TGF-β1 mAbs 1 day prior, and 6 days after infection. Expression of the indicated genes, normalized to b-actin expression, as measured by RT-qPCR in colon samples. Horizontal bars represent mean value ± sem per group. Data is representative of two independent experiments.

**Supplementary Fig. 3 Specific deletions of *Garp* in Tregs, B cells or platelets do not modify total serum Ig levels**

**a** Splenocytes from 3-month-old mice of the indicated genotypes were isolated and stimulated *in vitro* with anti-CD3/28 coated beads during 24 hours, then analyzed by flow cytometry. Each point indicated proportions of GARP positive cells in indicated cell type for one mouse (n=5-7). **b-d** Genetically modified C57BL/6 mice were bled and concentration of total Igs in serum was measured by ELISA. Data are pooled from two experiments and each point indicates concentration of total Ig subtype for one mouse (n=5-18). Horizontal bars represent mean ± SEM. Statistical analysis was performed with Mann-Whitney unpaired t-test and p-value is shown if it is lower than 0.5.

**Supplementary Fig. 4 Specific deletion of *Garp* in Tregs does not modify serum specific OVA-Ig levels**

Three-month-old Foxp3^cre^Garp^wt/wt^ and Foxp3^cre^Garp^fl/fl^ mice were immunized *i.p.* with OVA protein (100 μg) with alum. After 9 days, OVA protein (100 μg) in PBS was injected *i.p.* as boost. Blood was collected on day 16 to measure anti-OVA Igs in serum by ELISA. Data points represent values in individual mice. Horizontal lines indicate mean ± sem (n=2-7 mice per group). Mann-Whitney unpaired *t-*test was used to compare the two groups of immunized mice; p-values were higher than 0.5.
